# Morphological and Photosynthetic Response to High and Low Irradiance of *Aeschynanthus longicaulis*

**DOI:** 10.1155/2014/347461

**Published:** 2014-06-30

**Authors:** Qiansheng Li, Min Deng, Yanshi Xiong, Allen Coombes, Wei Zhao

**Affiliations:** ^1^School of Ecology, Shanghai Institute of Technology, Shanghai 201418, China; ^2^Shanghai Chenshan Plant Science Research Center, Chinese Academy of Sciences, Shanghai Chenshan Botanical Garden, Shanghai 201602, China; ^3^Herbarium and Botanic Garden, Benemerita Autonomous University of Puebla, 72000 Puebla, PUE, Mexico

## Abstract

*Aeschynanthus longicaulis* plants are understory plants in the forest, adapting to low light conditions in their native habitats. To observe the effects of the high irradiance on growth and physiology, plants were grown under two different light levels, PPFD 650 *μ*mol*·*m^–2^ *·*s^–1^ and 150 *μ*mol*·*m^–2^ *·*s^–1^ for 6 months. Plants under high irradiance had significantly thicker leaves with smaller leaf area, length, width, and perimeter compared to the plants grown under low irradiance. Under high irradiance, the leaf color turned yellowish and the total chlorophyll decreased from 5.081 mg*·*dm^−2^ to 3.367 mg*·*dm^−2^. The anthocyanin content of high irradiance leaves was double that of those under low irradiance. The plants under high irradiance had significantly lower A_max_ (5.69 *μ*mol*·*m^–2^ *·*s^–1^) and LSP (367 *μ*mol*·*m^–2^ *·*s^–1^) and higher LCP (21.9 *μ*mol*·*m^–2^ *·*s^–1^). The chlorophyll fluorescence parameter *F*_*v*_/*F*_*m*_ was significantly lower and NPQ was significantly higher in high irradiance plants. RLCs showed significantly lower ETR_max⁡_ and *E*_*k*_ in plants under high irradiance. It can be concluded that the maximum PPFD of 650 *μ*mol*·*m^–2^ *·*s^–1^ led to significant light stress and photoinhibition of *A. longicaulis*.

## 1. Introduction

*Aeschynanthus* Jack (Gesneriaceae) comprises approximately 160 species distributed from Sri Lanka and India through southern China and Southeast Asia to New Guinea and the Solomon Islands. The estimated number of species will undoubtedly change over time as more species are revised [1].* Aeschynanthus* plants are noted for their brilliant red, orange, or yellow tubular flowers that often appear in large terminal clusters. The interesting shape of the calyx and emerging bud has given some of them the common name of “lipstick plant.” The plants are usually epiphytic, shrubby, climbing, or trailing in habit, with dark green or mottled waxy leaves. They can be grown in a hanging pot or basket at home or outdoors. The best known species is* A. pulcher* (Blume) G. Don. There are also some cultivars which are popular in horticulture, for example,* Aeschynanthus* “Bali,” “Red Cascade,” “Hot Flash,” “Rigel,” and “Big Apple,” with different flower colors [2]. It is estimated that more than 50 species and/or cultivars are marketed for ornamental purposes.* Aeschynanthus* plants are now among the most popular hanging basket and potted flowering plants in the floriculture industry [3].

*A. longicaulis* Wall. ex R.Br. is native to South Yunnan of China, Vietnam, Thailand, and Malaysia. It produces clusters of orange flowers against trailing stems of dark green leaves, from summer to winter. The back of the leaf is mottled. Stems extend to 40 cm, glabrous. Leaves are opposite; petiole is absent or reaching 5 mm; leaf blade is elliptic to lanceolate or oblanceolate, 6.5–12 × 2.1–3.3 cm, papery to leathery, glabrous, adaxially drying wrinkled, abaxially sparsely punctate, base cuneate, margin crenulate and undulate, and apex acuminate; lateral veins are indistinct. With its trailing or pendulous stems and attractive leaf color, it can be used as a hanging basket plant [4, 5].

*Aeschynanthus* species are easy epiphytes to cultivate and propagate. They can be grown in hanging baskets with a free draining and open compost consisting of bark, perlite, vermiculite, and charcoal, which allows water to pass through easily but can hold enough moisture for plant growth [6]. They generally grow all year round in the greenhouse kept at 18–24°C. Among environmental factors, light, both quantity and quality have a great impact on the growth and flowering of* Aeschynanthus*. Gertsson reported that* Aeschynanthus* was a long-day plant [7], while Whitton et al. further proved that temperature interacted with photoperiod to influence flowering of hybrid “Koral”; light quantity may also have effects on flowering [8, 9].

*Aeschynanthus *plants are understory plants adapting to low light conditions in their native habitats [10]. Low irradiance generally leads to larger leaves with reduced thickness, stomatal density, and conductive tissue per unit leaf area [11]. Tree canopy responses to low light include increased internode length and reduced leaf area index. Plants also exhibit numerous physiological adaptations to low irradiance, including increased quantum yield and reduced dark respiration, light compensation, and saturation points [12]. However, low light grown plants have been frequently reported to be more susceptible to photoinhibition than high light grown plants [13, 14]. To cope with high light stress, plants may alter the pigments, structure, and orientation of leaves, especially in shade plants [15]. Thus, the appearance of the foliage can change greatly, affecting the aesthetic value. During our cultivation practice, we have noticed that the growth, leaf color, and morphological traits of* A. longicaulis* showed great differences when the plants were exposed on the greenhouse bench without additional interior shading. The considerable changes of leaf color and morphology are caused by the response of photosynthetic pigments and apparatus. These changes of physiological and morphological traits that allow the shade-adapted plants to thrive in high light might be detrimental to the photosynthetic apparatus and finally affect their growth. We hypothesized that, as being understory plants native to low light habitats,* A. longicaulis* may suffer photoinhibition or even photodamage when the plants are grown under high light intensity, although they may develop various strategies to cope with the high light stress, including changing the structure, pigments, and photosynthetic apparatus for photoprotection. The aims of this study were to compare the leaf anatomical, morphological, and photosynthetic differences of* A. longicaulis* grown under high and low light intensity in the greenhouse and reveal if photoinhibition occurs by measuring traditional photosynthetic light response curves and chlorophyll fluorescence.

## 2. Materials and Methods

### 2.1. Plant Material and Experiment Design

The stock plant was obtained from Shanghai Chenshan Botanical Garden and propagated by single node cutting. The voucher specimen DM 6917 is deposited in CSH. Plants with a single stem of about 10–12 cm in length grown from cuttings were planted in 12 cm diameter plastic pots. Each pot contained 3 plants. Plants were divided into two groups for low and high irradiance treatments in a shaded greenhouse, with 25 pots in each group. Each pot was filled with a peat-based potting mix (70% peat, 20% perlite, and 10% vermiculite based on volume) and top dressed with 5 g 18N-6P_2_O_5_-12K_2_O controlled release fertilizer. The maximum light intensity of the high irradiance treatment was maintained under 650 *μ*mol*·*m^−2^ *·*s^−1^ of PPFD by internal and external shading of the greenhouse. The maximum light intensity of the low irradiance treatment was maintained under 150 *μ*mol*·*m^−2^ *·*s^−1^ of PPFD by extra shading on the bench. All plants were grown for 6 months from April to September.

### 2.2. Leaf Morphology and Anatomy

At the end of the experiment, 25 fully expanded mature leaves from different pots of each treatment were collected for measurements. The stem length and node number were recorded and the average node length was calculated. Leaf thickness was measured with a micrometer. Leaf area, leaf perimeter, leaf length, and leaf width were measured using a portable leaf area meter (Yaxin-1241, Beijing). Leaf fresh mass (FM) and dry mass (DM) of each single leaf were measured using a balance, and specific leaf weight (SLW) was calculated on a dry mass basis by dividing the leaf area of one leaf by its dry mass. The leaf water content (LWC), as a percentage of fresh mass, was calculated according to the following formula: LWC (%) = 100 ((FM − DM)/FM).

Freehand cross-sections of fresh, unstained leaves were prepared by using an ethanol-cleaned razor blade and cutting from the midrib to the leaf margin. Sections were observed using an Olympus BX51 optical microscope. Photomicrographs were taken using a Canon G12 digital camera.

### 2.3. Pigments Assay

Chlorophyll was extracted in 95% ethanol. Four leaf discs were punched with a 6 mm diameter puncher (having a total surface of 1.12 cm^2^) and placed in a vial with 5 mL ethanol. Vials were kept in the dark at 4°C in a refrigerator for 20 hours with occasional shaking. The amounts of Chl (a + b) and carotenoid were measured spectrophotometrically [16]. Another four leaf discs were extracted with 1% (w/v) HCl in methanol, and the anthocyanin contents were assayed spectrophotometrically. The relative amounts of anthocyanin were expressed by *A*_530_ − 0.25*A*_657_ [17]. Absorbance was measured with a Hitachi U-5100 UV-visible spectrophotometer.

### 2.4. Stomatal Density

The traditional method of making epidermal imprints using clear nail polish was used to measure the stomatal density [18]. A thick layer of clear nail polish was brushed onto the lower epidermis of each leaf, while it was still on the living stem in the pots. Once dried, the nail polish was peeled off the back of the leaf and placed on a slide marked in millimeters. The impression was then viewed under 200x magnification using a light microscope. A representative section was chosen and the stomatal densities were calculated. Five independent counts were carried out on each leaf. Six individual leaves were sampled from the second node on the stem from different pots of each treatment.

### 2.5. Photosynthetic Light Response Curve

The photosynthetic light response curves were measured in the morning in September 2013 using a Li-6400 portable photosynthesis meter (Li-COR Bioscience, Lincoln, NE) on the newest developed mature leaves of each treatment. The range of PPFD (photosynthetic photon flux density) was set at 1500, 1000, 500, 250, 120, 60, 30, 15, and 0 *μ*mol*·*m^−2^ *·*s^−1^ using the Li-6400-02B light source. The CO_2_ concentration was kept stable around 380 mmol*·*mol^−1^, the rate of air flow was maintained at 300 mmol*·*s^−1^, and the leaf chamber (2 × 3 cm) temperature was set at 28°C. Curve-fitting software (Sigma Plot for Windows 11.0; Systat Software, Richmond, CA) was used to analyze the light responses using a three-component exponential function equation *A* = *a*(1 − *e* ^−*bx*^) + *c*, where *A* = net photosynthetic rate, *x* = PPFD, and *a*, *b*, and *c* were parameters estimated by the nonlinear regression [19]. Light-saturated photosynthesis rate *A*_sat⁡_ was calculated as *a* + *c*, and the quantum yield of photosynthesis (*A*_qe_) was calculated as the initial slope at *A* = 0 [calculated as *b* (*a* + *c*)]. The light compensation point (LCP) was determined by solving this equation for PPFD at *A* of 0 mmol*·*m^−2^ *·*s^−1^. The light saturation point (LSP) was determined by the PPFD at which *A* was 99% of the light-saturated net photosynthesis [20, 21].

### 2.6. Chlorophyll Fluorescence Measurements

Leaves were dark adapted with leaf clips for 30 min before measurement of the chlorophyll fluorescence parameters; the induction curve (slow kinetics) for photosystem II (P680) was measured by using a PAM-2500 chlorophyll fluorescence measuring device (Walz, Effeltrich, Germany). Values of maximum photochemical quantum yield of PS II (*F*_*v*_/*F*_*m*_), nonphotochemical quenching (NPQ), and nonregulated energy dissipation (NO) were calculated. After that, the rapid light curves were measured using a preinstalled software routine, where the actinic illumination was incremented in 10 steps from 5 to 1303 *μ*mol*·*m^−2^ *·*s^−1^, 30 s for each level. RLC was fitted and the cardinal points *α* (the initial slope of the rapid light curve which is related to the maximal quantum yield of PS II electron), ETR_max⁡_ (relative maximum electron transport rate), and *E*_*k*_ (minimum saturating irradiance) were derived by the PamWin 3.12 (Walz, Effeltrich, Germany).

### 2.7. Data Analysis

Data were analyzed using the IBM SPSS statistics 19 (SPSS Inc., 2010). All data were subjected to analysis of variance and *t*-test was employed to analyze the differences.

## 3. Results

### 3.1. General Plant and Leaf Characteristics

**Figure 1:**
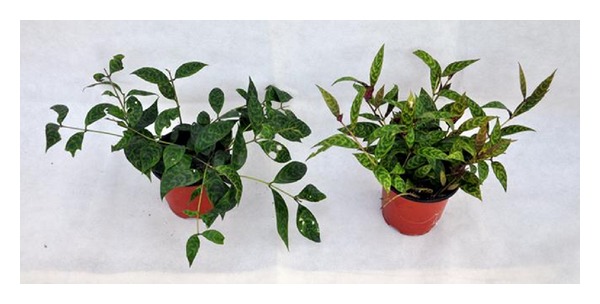
The different appearance of whole plants grown under low (left) and high (right) light intensity (pot diameter 12 cm).

Whole plants grown under high light intensity were more compact with more lateral shoots but developed a yellowish appearance (Figure 1).

**Figure 2:**
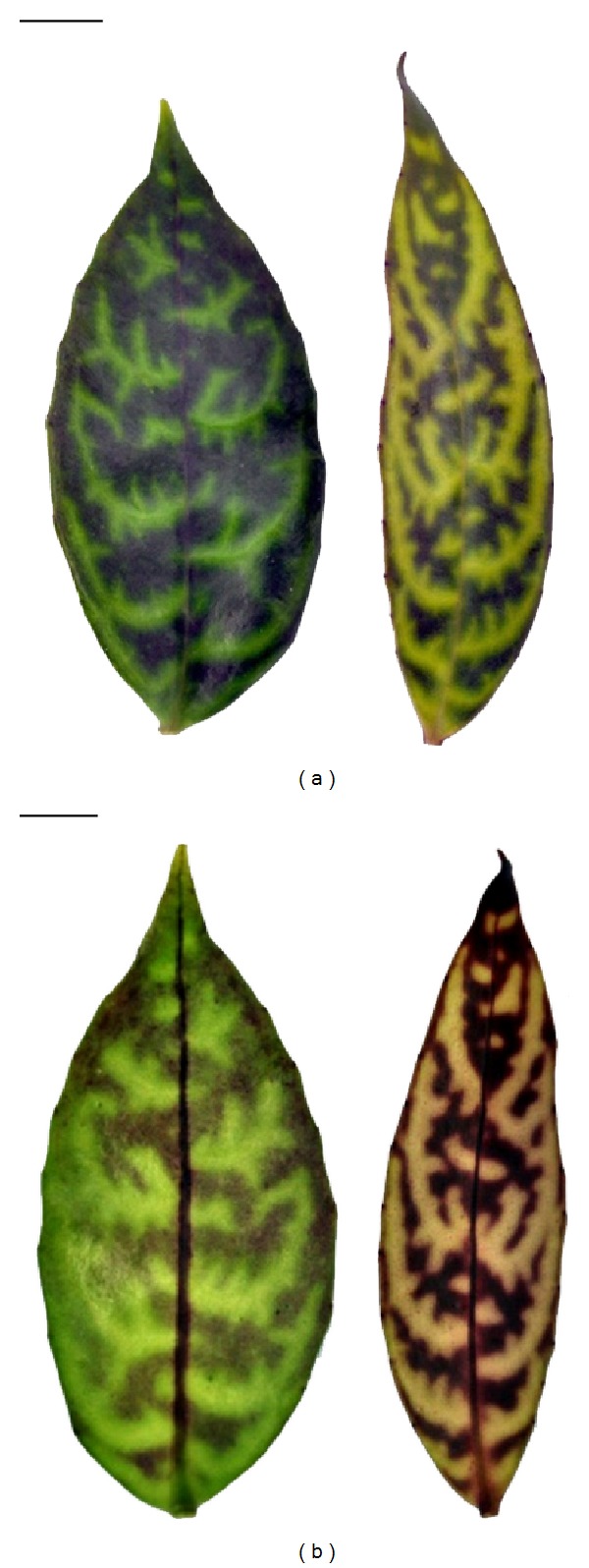
The morphology of upper and lower surfaces of single leaves from plants grown under low and high light intensity. (a) Upper surface of leaf under low (left) and high (right) irradiance; (b) lower surface of leaf under low (left) and high (right) irradiance. Bar = 1 cm.

The average internode length of pants under low irradiance was 3.8 cm, significantly longer than that of plants under high irradiance (2.9 cm). The leaves grown under high irradiance were typically narrower with a thicker appearance as compared to the leaves under low irradiance. Leaves under high irradiance had a significantly smaller leaf area, length, width, and perimeter length compared to the thinner leaves of plants grown under low irradiance. The average leaf area of a single leaf and the specific leaf weight of leaves under high irradiance were almost double those of the shade leaves (Figure 2, Table 1). The leaves of high irradiance also possessed lower relative water content (Table 1). The stomatal density of the leaves under high irradiance was 23% greater than that of those under low irradiance.

### 3.2. Leaf Anatomy

**Figure 3:**
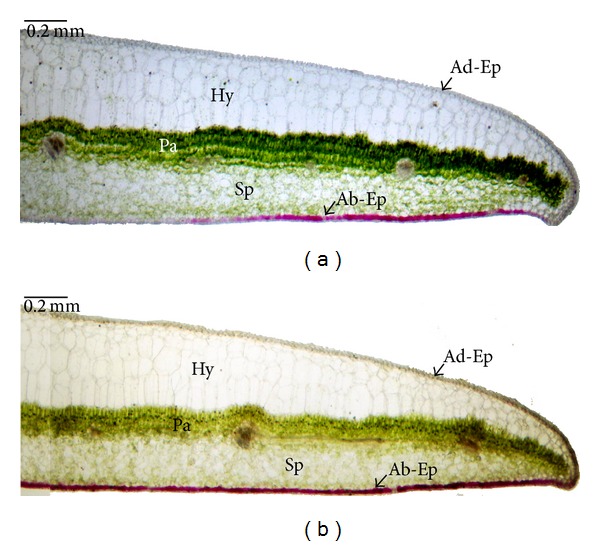
Cross-section of* A. longicaulis* leaf showing the distribution of chlorophyll in the palisade parenchyma and anthocyanin in the lower epidermal cells ((a) low light; (b) high light). The section has been taken across the middle of the leaf blade. Ad-Ep: leaf adaxial epidermis, Ab-Ep: leaf abaxial epidermis, Hy: hypodermis, Pa: palisade tissue, Sp: spongy parenchyma.

At the light microscope level no visual differences in the structure of internal leaf tissues were observed between the fresh leaves under high and low irradiance. In the transverse leaf section there is a layer of cells that appears to be a typical epidermis, under which a multiple hypodermis of 3–5 layers (from the margin to the middle of the leaf) of giant, empty-looking, parallel cells is found (Figure 3). These cells also vary in size, with the internal ones being larger and almost totally filled by the vacuole. The thick multiple-layered hypodermis of the plants grown under two distinct light intensities was of similar size and responsible for the majority (about 50%) of the thickness of the leaf lamina. The palisade cells were in 3-4 layers. The treatments did not alter these features, but in the leaves under low irradiance, the palisade cells were more compact and contained more and greener chloroplasts (Figure 3(a)), while in the leaves under high irradiance, they appeared more loosely arranged and yellowish (Figure 3(b)). A few chloroplasts were scattered in the spongy parenchyma. Most parts of the lower epidermis were red in color, especially in the high irradiance leaves, indicating that the anthocyanin was concentrated in the lower epidermal cells.

### 3.3. Leaf Pigments

The differences in chlorophyll (Chl) and total carotenoid (Cars) contents between high and low irradiance leaves are summarized in Table 2. The Chl a, Chl b, and Chl (a + b) levels based on leaf area were significantly lower in leaves under high irradiance compared to those of leaves under low irradiance (about 1/3 lower), but the differences of Chl a/b ratio and carotenoid content were not statistically significant. However, the anthocyanin content per leaf area was doubled in the sun leaves compared to that of shade leaves. According to microscopic observations of cross-sections of the leaf lamina, anthocyanin was predominantly located in the lower epidermis and the lowest layer of spongy mesophyll cells. The chlorophyll was mostly concentrated in the 3–5 palisade mesophyll layers and sporadically distributed in the spongy mesophyll cells.

### 3.4. Light Response Curve of Photosynthesis

**Figure 4:**
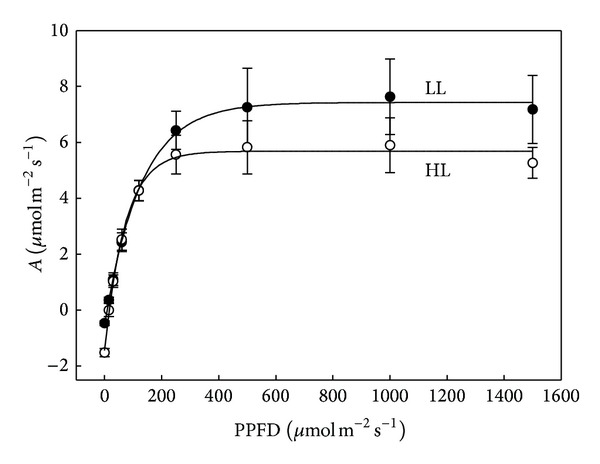
Light response curves of net assimilation rate (*A*) for* A. longicaulis* plants grown under high light (HL) and low light (LL). Data are means ± SE (*n* = 5).

The net photosynthetic rates (*A*) of* A. longicaulis* increased rapidly as PPFD increased from 0 to 250 *μ*mol*·*m^−2^ *·*s^−1^ and then remained stable (Figure 4). The light response curves revealed that the maximum photosynthetic rate of the plants under low irradiance was greater than that of the plants under high irradiance. Leaves under low irradiance had higher *A*_max⁡_ (7.49 *μ*mol*·*m^−2^ *·*s^−1^) and LSP (652 *μ*mol*·*m^−2^ *·*s^−1^) and lower LCP (9.1 *μ*mol*·*m^−2^ *·*s^−1^), while the plants under high irradiance had significantly lower *A*_max⁡_ (5.69 *μ*mol*·*m^−2^ *·*s^−1^) and LSP (367 *μ*mol*·*m^−2^ *·*s^−1^) and higher LCP (21.9 *μ*mol*·*m^−2^ *·*s^−1^). However, the quantum yield (*A*_qe_) of plants under high irradiance was still higher than that of the low irradiance (0.0789 versus 0.0579 mol CO_2_/mol quantum) (Table 3).

### 3.5. Chlorophyll Fluorescence and RLC

Leaves exposed to high light showed a reduction in the maximum quantum yield of PS II (*F*_*v*_/*F*_*m*_), indicating that these leaves suffered photoinhibition or photodamage. In HL plants, significantly higher NPQ and qN (nonphotochemical quenching) were observed, with significant lower qP and qL (coefficient of photochemical quenching) values (Table 4), which indicated that more energy was dissipated as heat in HL leaves.

**Figure 5:**
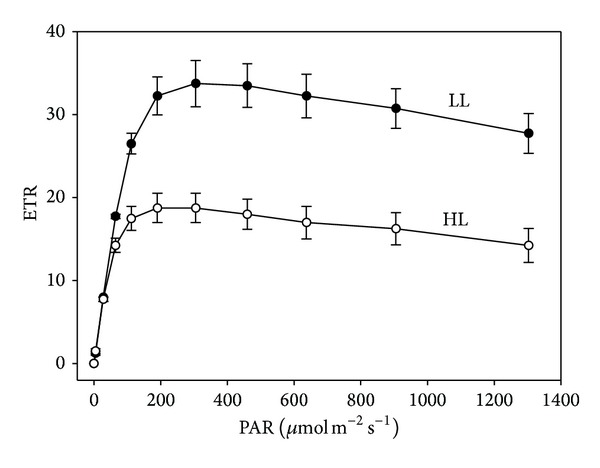
Rapid light curves of high light (HL) and low light (LL) grown* A. longicaulis* plants, where the relative electron transport rate (ETR) is plotted against the PAR irradiance. Data are means ± SE (*n* = 6).

In addition to the traditional light curves based on CO_2_ assimilation at different PAR levels, rapid light curves (RLCs) were measured by a PAM pulse modulation fluorometer. The rapid light curves, when ETR was plotted against PAR (Figure 5), were similar to a traditional CO_2_ assimilation based photosynthetic light curve (Figure 4). RLC showed that HL and LL grown plants had very similar initial increases in ETR (*α*), with HL samples being saturated at considerably lower maximum rates (Figure 5). The ETR_max⁡_ in the HL leaf was 42% lower than that in the LL leaf. The minimum saturating irradiance (*E*_*k*_), which is related to quenching, was also significantly decreased in the high light cultivated plants (Table 5).

## 4. Discussion

*A. longicaulis* is a typical shade-tolerant plant. When it was grown under high irradiance, the plants may be under stress and disordered physiology. The whole plant growing under high irradiance in this study turned yellowish and thus influenced the ornamental appearance. The stems were more fragile. As a response to high light stress and to reduce whole light interception, plants of* A. longicaulis* showed reduced specific leaf area, increased leaf thickness, and reduced chlorophyll content. In addition, plants grown in high light intensities were more erect compared to those grown in low light which showed a more creeping habit. These responses are similar to those found in other plants [12].

It is already known that the hypodermis together with the epidermis functions in focusing and concentrating light [22], facilitating its penetration into leaves. In this study, we found that there were 3–5 layers (from the margin to the middle of the leaf) of giant, lens-like, parallel hypodermis cells without chloroplasts, both in the leaves of high irradiance and low irradiance. These cells store water, preventing desiccation to which epiphytic plants can be prone [1]. This special structure is also very important for shade-adapted plants to keep a higher photosynthetic rate at extremely low light levels by both the focusing effects and reducing leaf reflectance [23]. The thick epidermal tissue may also function to protect internal damage to the mesophyll by UV-B radiation, as in* Peperomia* [24]. The same structure found in both treatments indicated that the short-term high irradiance treatment did not change the inherent leaf structure of* A. longicaulis.*

Botanists noticed that anthocyanin production rises when a plant is subjected to low temperatures and high light conditions. The popular explanation was that the anthocyanin protects the photosynthetic structures against intense sunlight and helps warm leaves by increasing their rates of metabolism. It was believed that anthocyanin acts as sun screener and antioxidant to protect plants against light damage [25]. However, anthocyanin must be held in the upper epidermis and/or hypodermis to screen UV*-*B, in the mesophyll to protect chloroplasts from photoinhibition or in the lowermost tissues to enhance light capture by internal reflection [26]. In this study, the anthocyanin content of high irradiance leaves was double that of low irradiance leaves, and the anthocyanin was concentrated in the lower epidermis of* A. longicaulis* leaves (Figure 3). This indicates that the doubled contents of anthocyanin in leaves of high irradiance most likely acted as an antioxidant by scavenging free radicals instead of direct shielding. However, the data of photosynthesis and chlorophyll fluorescence show that this could not completely alleviate the stress of photoinhibition under high irradiance even though the leaves of* A. longicaulis* grown under high irradiance had double the anthocyanin concentration of leaves grown under low irradiance. Similar results were reported in* Pelargonium* ×*hortorum* [27]. Also, there was no difference in carotenoid content of the high and low light grown* A. longicaulis* in our experiment, though it is believed that carotenoids play an important role in protection of photosynthesis by losing excitation energy through the xanthophyll cycle [28].

The photosynthetic pigment, chlorophyll, decreased with high irradiance and increased with low irradiance in* A. longicaulis*. High irradiance resulted in a yellowish leaf surface appearance and the same color difference was found in the leaf sections. These results are consistent with those found in other plants [29]. The chlorophyll a/b ratios of both treatments were very low, which is the typical adaptation of shade plants.

In general, plants grown under higher PPFD have high light saturation points because of the higher level of enzymes for carboxylation and electron transport [30]. Shade-tolerant plants typically have low photosynthetic rates but have the ability to efficiently capture and use light energy, usually through increased chlorophyll concentration and low Chl a/b ratio [12]. For most plants, such as* Pachira aquatica* [21] and* Carya illinoinensis* [31], shading generally induced lower *A*_max⁡_ and *L*_cp_. However, exposure of leaves to excessive light is a well-known cause of photoinhibition [32]. It is associated with a decrease in the ratio of variable to maximal fluorescence (*F*_*v*_/*F*_*m*_) [14] and is manifested by a reduction in photosynthesis.

As a shade plant,* A. longicaulis* grown under high irradiance had a higher LCP but lower *A*_max⁡_ and LSP. It was apparent that photoinhibition occurred when the plants were grown at the maximum PPFD 650 *μ*mol*·*m^−2^ *·*s^−1^, as indicated by a significant lower *F*_*v*_/*F*_*m*_ value. The *F*_*v*_/*F*_*m*_ is widely used as an indicator of photoinhibition of photosynthesis and can be easily determined in dark adapted leaves [33]. The *F*_*v*_/*F*_*m*_ of* A. longicaulis* under low irradiance was 0.786; however when they were grown under high irradiance for 6 months, the value decreased to 0.601 in our experiment.

Photon energy captured by chlorophyll a molecules can either drive photosynthesis (photochemical quenching, qP), be emitted as fluorescence, or be converted to heat (nonphotochemical quenching, qN and NPQ). Heat dissipation is linked to the xanthophyll cycle, which protects the photosynthetic apparatus from high light damage. NPQ is more sensitive and often used as an indicator of the excess-radiant energy dissipation to heat in PSII antenna complexes [34]. In this study, significantly higher NPQ was observed in plants under higher light. The increased NPQ could dissipate the partial excess incoming photon energy, preventing damage to the photochemical pathway, before the energy is accumulated as reactive intermediate substances in the photosynthetic chain [35].

Besides the traditional light response curve based on CO_2_ assimilation, the RLC obtained by plotting rETR against PAR can provide a reliable assessment of photosynthetic function of irradiance [36]. The electron transport rate (ETR) was found to be closely related to the photosynthetic activity based on CO_2_ uptake [37]. The greatly reduced ETR_max⁡_ and *E*_*k*_ values further supported the fact that photoinhibition occurred in plants of* A. longicaulis* grown under high irradiance, although the increased NPQ may partially dissipate the excess photon energy.

It can be concluded from this study that when the shade-tolerant plant* A. longicaulis* was grown under high light intensity there were significant changes in growth, whole plant appearance, leaf structure, leaf pigments, and photosynthesis. All the data from traditional light response curves and chlorophyll fluorescence support the fact that the maximum PPFD of 650 *μ*mol*·*m^−2^ *·*s^−1^ leads to significant light stress and photoinhibition. Further detailed research is necessary to evaluate the most suitable growth light level between PPFD of 650 *μ*mol*·*m^−2^ *·*s^−1^ and 150 *μ*mol*·*m^−2^ *·*s^−1^.

## Figures and Tables

**Table 1:** Leaf characteristics of plants grown under high and low light.

Leaf characteristics	High light	Low light	Significance
Thickness (mm)	1.12 ± 0.04	0.86 ± 0.03	∗∗∗
Area (mm^2^)	812.0 ± 29.33	1524.5 ± 70.42	∗∗∗
Perimeter (mm)	163.4 ± 7.33	182.8 ± 3.82	∗
Length (mm)	73.2 ± 0.94	83.8 ± 1.73	∗∗∗
Width (mm)	18.0 ± 0.49	28.7 ± 0.71	∗∗∗
SLW (g/m^2^)	340.0 ± 9.92	171.5 ± 8.09	∗∗∗
Water content (%)	93.0 ± 0.11	95.0 ± 0.17	∗∗∗
Stomatal density (number/mm^2^)	86.9 ± 2.4	70.7 ± 1.7	∗∗∗

Data are means ± SE (*n* = 25); ^∗,∗∗,∗∗∗^indicate significant difference at *P* < 0.05, 0.01, and 0.001, respectively.

**Table 2:** Contents of leaf pigments of plants grown under high and low light.

Pigment content	High light	Low light	significance
Chl a (mg*·*dm^−2^)	2.030 ± 0.130	3.026 ± 0.108	∗∗∗
chl b (mg*·*dm^−2^)	1.337 ± 0.087	2.055 ± 0.071	∗∗∗
Chl (a + b) (mg*·*dm^−2^)	3.367 ± 0.216	5.081 ± 0.178	∗∗∗
chl a/b	1.522 ± 0.023	1.473 ± 0.012	
Carotenoids (mg*·*dm^−2^)	0.388 ± 0.026	0.392 ± 0.022	
Anthocyanin (*A*_530_)	0.300 ± 0.028	0.143 ± 0.019	∗∗∗

Data are means ± SE (*n* = 9); ***indicated significant difference at *P* < 0.001.

**Table 3:** Maximum net photosynthetic rate (*A*_max⁡_), quantum yield (*A*_qe_), light compensation point (LCP), and light saturation point (LSP) of *A. longicaulis *plants grown under high and low light.

	*A*_max⁡_ (*μ*mol*·*m^−2^ *·*s^−1^)	*A*_qe_ (mol*·*CO_2_/mol*·*quantum)	LSP (*μ*mol*·*m^−2^ *·*s^−1^)	LCP (*μ*mol*·*m^−2^ *·*s^−1^)
Low irradiance	7.49 ± 1.23	0.0601 ± 0.013	652.4 ± 24.6	9.1 ± 1.14
High irradiance	5.69 ± 0.85	0.0786 ± 0.019	366.8 ± 12.65	21.9 ± 4.14
Significance	∗∗	∗	∗∗∗	∗∗∗

Data are means ± SE (*n* = 5); ^∗,∗∗,∗∗∗^indicate significant difference at *P* < 0.05, 0.01, and 0.001, respectively.

**Table 4:** Selected parameters of chlorophyll fluorescence measured on dark adapted leaves when grown under high and low light conditions.

	High light	Low light	Significance
*F*_*v*_/*F*_*m*_	0.601 ± 0.012	0.786 ± 0.011	∗∗
NPQ	0.935 ± 0.066	0.326 ± 0.044	∗∗∗
qN	0.603 ± 0.021	0.296 ± 0.029	∗∗
qP	0.637 ± 0.016	0.880 ± 0.013	∗∗
qL	0.435 ± 0.017	0.719 ± 0.023	∗∗

Data are means ± SE (*n* = 9); ^∗∗,∗∗∗^indicate significant difference at *P* < 0.01 and 0.001, respectively.

**Table 5:** Cardinal points of the rapid light curves measured for leaves of *A. longicaulis* grown under high and low light conditions.

	High light	Low light	Significance
*α*	0.4581 ± 0.0214	0.4436 ± 0.0175	
ETR_max⁡_	20.09 ± 3.12	33.62 ± 3.56	∗∗
*E*_*k*_	43.89 ± 5.23	76.58 ± 6.87	∗∗

Data are means ± SE (*n* = 6); **indicated significant difference at *P* < 0.01.

## References

[1] Middleton DJ (2007). A revision of *Aeschynanthus* (Gesneriaceae) in Thailand. *Edinburgh Journal of Botany*.

[2] The Gesneriad Society (2007). *How to Know and Grow Gesneriads*.

[3] Cui J, Chen J, Henny RJ (2009). Regeneration of *Aeschynanthus radicans* via direct somatic embryogenesis and analysis of regenerants with flow cytometry. *In Vitro Cellular and Developmental Biology: Plant*.

[4] Brickell C (2008). *A–Z Encyclopedia of Garden Plants*.

[5] Wang WC, Pan K, Li Z, Weitzman AL, Skog LE, Wu CY, Raven PH (1999). Gesneriaceae. *Flora of China*.

[6] Middleton DJ (2010). Aeschynanthus buxifolius. *Curtis's Botanical Magazine*.

[7] Gertsson UE (1987). Influence of light on flowering in *Aeschynanthus speciosus* Hook. *Journal of Horticultural Science*.

[8] Whitton B, Healy W, Roh M (1990). Flowering of *Aeschynanthus* “Koral” at fluctuating and constant temperatures. *Journal of the American Society for Horticultural Science*.

[9] Whitton B, Healy W, Roh M (1991). Flowering of *Aeschynanthus* ‘Koral’. *HortScience*.

[10] Burtt BL, Woods JB (1975). Studies in the Gesneriaceae of the old world, XXXIX: towards a revision of *Aeschynanthus*. *Notes from the Royal Botanic Garden, Edinburgh*.

[11] Stanton KM, Weeks SS, Dana MN, Mickelbart MV (2010). Light exposure and shade effects on growth, flowering, and leaf morphology of *Spiraea alba* du roi and *Spiraea tomentosa* L. *HortScience*.

[12] Taiz L, Zeiger E (2006). *Plant Physiology*.

[13] Chow WS (1994). Photoprotection and photoinhibitory damage. *Advances in Molecular and Cell Biology*.

[14] Demmig-Adams B, Adams WW (1992). Photoprotection and other responses of plants to high light stress. *Annual Review of Plant Physiology and Plant Molecular Biology*.

[15] Smith WK, Vogelmann TC (1997). Leaf form and photosynthesis. *Bioscience*.

[16] Lichtenthaler HK (1987). Chlorophylls and carotenoids—pigments of photosynthetic biomembranes. *Methods in Enzymology*.

[17] Mancinelli AL, Hoff AM, Cottrell M (1988). Anthocyanin production in chl-rich and chl-poor seedlings. *Plant Physiol*.

[18] Sampson J (1961). A method of replicating dry or moist surfaces for examination by light microscopy. *Nature*.

[19] Watling JR, Press MC, Quick WP (2000). Elevated CO_2_ induces biochemical and ultrastructural changes in leaves of the C4 cereal sorghum. *Plant Physiology*.

[20] Peek MS, Russek-Cohen E, Wait DA, Forseth IN (2002). Physiological response curve analysis using nonlinear mixed models. *Oecologia*.

[21] Li Q, Deng M, Chen J, Henny RJ (2009). Effects of light intensity and paclobutrazol on growth and interior performance of *Pachira aquatica* aubl. *HortScience*.

[22] Vogelmann TC, Martin G (1993). The functional significance of palisade tissue: penetration of directional versus diffuse light. *Plant, Cell & Environment*.

[23] Bone RA, Lee DW, Norman JM (1985). Epidermal cells functioning as lenses in leaves of tropical rain-forest shade plants. *Applied Optics*.

[24] Gausman HW, Rodriguez RR, Escobar DE (1975). Ultraviolet radiation reflectance, transmittance, and absorptance by plant leaf epidermises. *Agronomy Journal*.

[25] Lee DW, Gould KS (2002). Why leaves turn red. *The American Scientist*.

[26] Gould KS, Markham KR, Smith RH, Goris JJ (2000). Functional role of anthocyanins in the leaves of *Quintinia serrata* A. Cunn.. *Journal of Experimental Botany*.

[27] Liakopoulos G, Spanorigas I (2012). Foliar anthocyanins in *Pelargonium* × *hortorum* are unable to alleviate light stress under photoinhibitory conditions. *Photosynthetica*.

[28] Demmig-Adams B, Adams WW (1996). The role of xanthophyll cycle carotenoids in the protection of photosynthesis. *Trends in Plant Science*.

[29] Brand MH (1997). Shade influences plant growth, leaf color, and chlorophyll content of *Kalmia latifolia* L. Cultivars. *HortScience*.

[30] Callan EJ, Kennedy CW (1995). Intercropping Stokes aster: effect of shade on photosynthesis and plant morphology. *Crop Science*.

[31] Lombardini L, Restrepo-Diaz H, Volder A (2009). Photosynthetic light response and epidermal characteristics of sun and shade pecan leaves. *Journal of the American Society for Horticultural Science*.

[32] Powles SB (1984). Photoinhibition of photosynthesis induced by visible light. *Annual Review of Plant Physiology*.

[33] Baker NR (2008). Chlorophyll fluorescence: a probe of photosynthesis in vivo. *Annual Review of Plant Biology*.

[34] Demmig-Adams B, Adams WW, Barker DH, Logan BA, Bowling DR, Verhoeven AS (1996). Using chlorophyll fluorescence to assess the fraction of absorbed light allocated to thermal dissipation of excess excitation. *Physiologia Plantarum*.

[35] Ralph PJ, Polk SM, Moore KA, Orth RJ, Smith WO (2002). Operation of the xanthophyll cycle in the seagrass *Zostera marina* in response to variable irradiance. *Journal of Experimental Marine Biology and Ecology*.

[36] Ralph PJ, Gademann R (2005). Rapid light curves: a powerful tool to assess photosynthetic activity. *Aquatic Botany*.

[37] Beer S, Vilenkin B, Weil A, Veste M, Susel L, Eshel A (1998). Measuring photosynthetic rates in seagrasses by pulse amplitude modulated (PAM) fluorometry. *Marine Ecology Progress Series*.

